# Fluorescence-Guided Identification of the Thoracic Duct by VATS for Treatment of Postoperative Chylothorax: A Short Case Series

**DOI:** 10.3389/fsurg.2022.912351

**Published:** 2022-05-04

**Authors:** Francesco Londero, William Grossi, Massimo Vecchiato, Antonio Martino, Antonio Ziccarelli, Roberto Petri, Angelo Morelli

**Affiliations:** ^1^ Thoracic Surgery Unit, Cardiothoracic Department, S. Maria della Misericordia University Hospital, Udine, Italy; ^2^ General Surgery Department, S. Maria della Misericordia University Hospital, Udine, Italy

**Keywords:** chylothorax, thoracic duct, indocyanine green, VATS, thoracic surgery, case series

## Abstract

**Background:**

Chylothorax is a relatively rare complication after surgery of the mediastinum. The occurrence and the results of surgical treatment of this condition are difficult to foresee due to the wide heterogeneity in thoracic duct anatomy.

**Case summary:**

We report two cases of postoperative chylothorax treated with ligation by video-assisted thoracoscopic surgery (VATS). The first patient developed a massive left chylothorax shortly after discharge, following radical excision of a seminoma-involved left para-aortic lymphadenopathy. The second patient developed a high-output right chylothorax following VATS upper bilobectomy. In both cases, a surgical revision by VATS was performed. Inguinal injection of indocyanine green allowed an easy visualization of the lymphatic leakage point. In both cases, oral feeding was rapidly restarted after surgery. No recurrence of chylothorax was observed.

**Conclusion:**

The use of indocyanine green may greatly improve the identification of the thoracic duct during surgical ligation by VATS, with a favorable impact on the postoperative course and overall admission costs.

## Introduction

Chylothorax is an accumulation of a typical milky-appearance lymphatic fluid within the pleural cavity and represents an uncommon complication after surgical procedures involving the mediastinum. Indeed, the incidence of postoperative chylothorax ranges between 0.5% and 3% after esophagectomy (1) and between 0.25% and 3% after lung surgery with associated lymphadenectomy (2–4). This complication accounts for a prolonged length of stay, the need for taking further therapeutic measures, such as fasting and implementation with parenteral nutrition protocols, and increased overall admission costs. Furthermore, high-output chylothorax may lead to a severe risk of death if not timely treated (5). Therefore, in this case, an expeditious surgical exploration and ligation of the thoracic duct (TD) may be proposed with the aim of preventing further complications and reducing the admission time. However, the intraoperative identification of the TD and its leakage point may be troublesome, due to the wide heterogeneity in the duct anatomy, resulting in the displeasing eventuality of lymphatic leak persistence (6). We hereby report our experience with two cases of postoperative chylothorax treated with TD ligation by video-assisted thoracoscopic surgery (VATS), after inguinal injection of indocyanine green (ICG), to delineate the course of the TD and identify the leakage point.

## Case Description

Approval was waived by our local Ethical Committee due to the nature of the study. However, both patients signed an informed consent form for the use of their personal data for scientific purposes.

### Case 1

**Figure 1:**
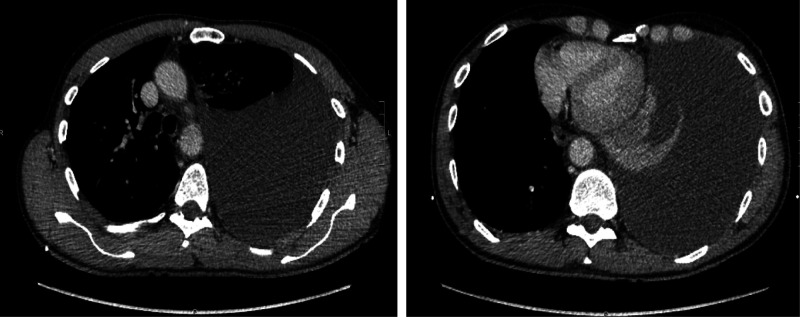
CT scan showing a uniform left massive pleural effusion.

A 56-year-old male patient underwent a left VATS radical excision of a 33-mm para-aortic metastatic lymph node (seminoma) at the level of the 8th thoracic vertebra, which at pathology report resulted as a testicular seminoma-involved lymph node. The postoperative course was uneventful, and the patient was discharged on postoperative day 3 in a good condition. Chest x-ray at discharge showed a mild left pleural effusion. Two days after discharge, he was admitted in the emergency department for left thoracic pain and severe dyspnea. Physical examination revealed normal blood pressure and a mild sinus tachycardia (96 beats per minute). Arterial oxygen saturation was 97% on room air. A first-assessment thoracic ultrasound revealed the presence of a massive anechoic left pleural effusion. Therefore, the patient was sent for an urgent chest computed tomography (CT) scan, which confirmed a massive homogeneous left effusion with contralateral mediastinal shifting (**Figure 1**). A left chest drain was promptly inserted, allowing a fractionated output of 3500 ml of serosanguinous fluid, which later separated in the form of a milky supernatant. Pleural fluid analysis revealed a triglycerides (TG) concentration of 521 mg/dl, consistent with chylothorax. The patient was set on immediate fasting and endovenous fluid replacement, following which his condition rapidly reverted to normality. The day after, he was scheduled for VATS ligation of the leakage point.

### Case 2

An 80-year-old male patient underwent a VATS upper bilobectomy and extended lymphadenectomy for a right lung T4N0 adenocarcinoma. On postoperative day 1, the chest tube drainage fluid turned milky, and the daily output reached nearly 1500 ml. Concurrently, the patient developed paroxysms of atrial fibrillation, low blood pressure, and general malaise. An endovenous hydration was started, electrolytes were corrected, and the patient was immediately set on absolute fasting. Pleural fluid analysis revealed a TG concentration of 838 mg/dl. After correcting for dehydration and electrolyte imbalance, the patient’s general conditions progressively improved, but the chest drain output was still as high as 500 ml/day while fasting.

On postoperative day 6, a VATS ligation of the TD was planned.

### Operative Technique

**Figure 2:**
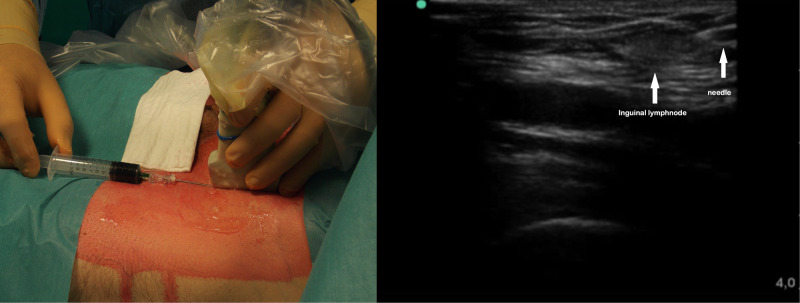
Ultrasound-guided inguinal lymph node administration of indocyanine green.

After general anesthesia induction, with the patient in the supine position, an ultrasound-guided inguinal lymph node injection of 10 ml saline with 0.5 mg/kg ICG solution (Infracyanine, SERB SAS, Paris, France) was bilaterally performed (**Figure 2**). The patient was, therefore, turned on his contralateral decubitus and operated under single lung ventilation. The VATS ports were reopened, and a 30° camera with a near infrared (NIR) light acquisition and overlay technology was employed (Karl Storz Image 1 S 4K ICG Rubina; Karl Storz SE & Co, Tuttlingen, Germany).

**Figure 3:**
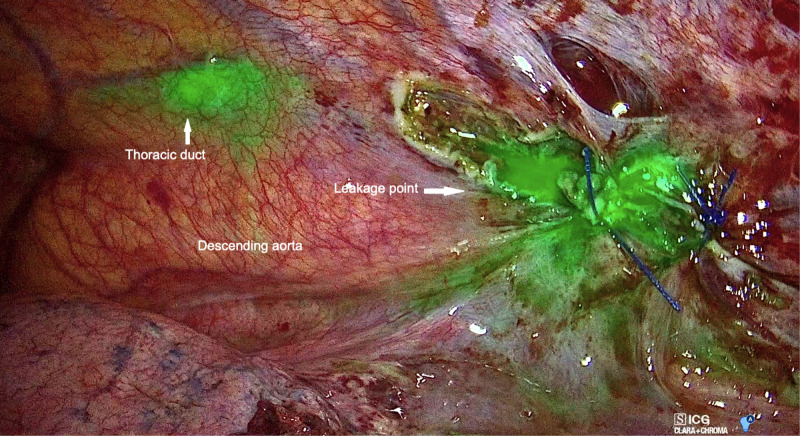
Left video-assisted thoracoscopic surgery (VATS) view showing fluorescent lymph spilling from the left para-aortic zone.

**Figure 4:**
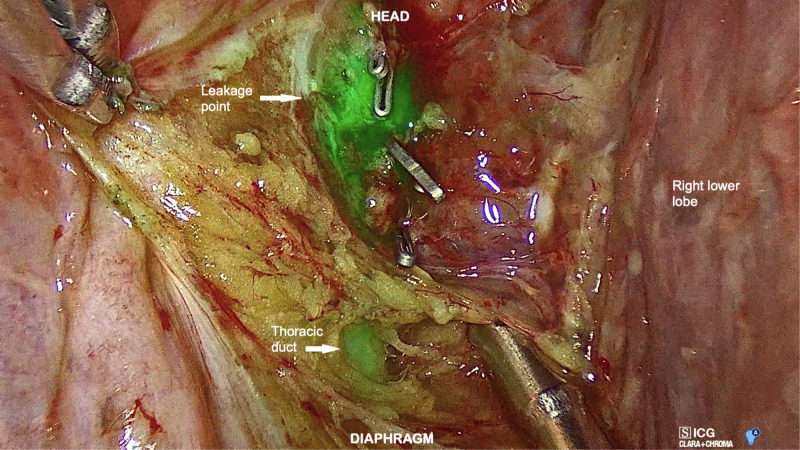
Right VATS view showing the thoracic duct and its leakage point in the low paraesophageal area.

In both cases, a clear visualization of the lymphatic structures and the leakage point was obtained within 20 min from inguinal injection. In Case 1, a dilated tributary of the TD was identified (**Figure 3**), while in Case 2, the leakage point was identified on the TD at the level of paraesophageal lymphadenectomy (**Figure 4**). In both cases, a manual ligation at the leakage point was performed, and a definitive closure of the lymphatic structure was observed due to the absence of further fluorescent lymph spilling.

### Outcomes

The duration of the thoracoscopic phase was 60 and 65 min, respectively. The fluorescent contrast was visible throughout the thoracoscopic phase. No significant blood loss or intraoperative complication was observed in both cases. With the aim of avoiding excessive stress on the ligatures and reducing the risk of chylous leak recurrence, oral feeding was prudentially restarted under a low lipidic content diet on postoperative days 5 and 7, respectively. After ascertaining the absence of lymphatic fistula recurrence, the chest drain was removed the following day. Both patients recovered with no further adverse event and were discharged in good overall condition on postoperative days 7 and 12, respectively. Chest x-rays performed at 7 and 30 days after discharge ruled out the recurrence of pleural effusion. At 6 months’ follow-up appointment, both patients made a complete recovery to active life with no limitation in their daily activities.

## Discussion

Chylothorax is a potentially life-threatening complication of mediastinal lymphadenectomy (5). It has been acknowledged that in the early stages, a conservative treatment, consisting of absolute fasting and parenteral nutrition for two weeks, may be proposed for low-output lymphatic fistulas (7). However, this might not be effective in resolving the chylous leak: in their recent series, Yang et al. reported a 82% rate of success of conservative treatment in patients with postoperative chylothorax, and, although a precise clinical description of successful cases was not offered, all failures occurred in patients with high-output fistulas (7). Therefore, under these circumstances, an expeditious treatment is a reasonable option, in order to prevent further deleterious evolutions. Indeed, dehydration, electrolyte imbalance, and malnutrition may quickly aggravate patients’ postoperative course and prove fatal in up to 30% of cases, if not properly treated (8).

With the rationale of reducing patients’ exposure to additional risks, a non-surgical therapeutic option is represented by lymphangiography. Indeed, along with giving important information on the anatomy of the lymphatic structures in view of a surgical ligation (6), this may also represent a preliminary step for percutaneous embolization of the cisterna chyli (9). This treatment is characterized by a low incidence of adverse events, and its effectiveness can be as high as 90% when liquid embolic agents and coils are used for occluding the cisterna chyli (9). However, in our cases, we preferred to use the surgical option due to the high output of the lymphatic fistula and with the perspective of confirming the reliability of this technique. Moreover, the therapeutic choice was shared with the patients after illustrating the respective pros and cons of both conservative and invasive modalities.

The surgical treatment of this condition relies on the ligation of the TD upstream of the leakage point. However, the identification of this anatomic structure may be quite challenging due to the high variability of its course and the presence of traumatized tissues around the spilling area (10). Several modalities have been proposed to facilitate its recognition, such as lymphangiography or preoperative administration of fat meals, to which methylene blue may be added (11). Nevertheless, the duct might not be recognized, and, under these circumstances, an en-mass ligation of the supra-diaphragmatic region between descending thoracic aorta and azygos vein may be performed, with a success rate that may reach 90% (12).

The thoracoscopic approach, along with the established advantages in terms of lower surgical invasiveness, allows a magnified vision of anatomical structures, which may aid in the identification of the TD. In recent years, the advisability of using this approach has been reinforced by the introduction of NIR light acquisition techniques. Indeed, the administration of ICG into the inguinal lymph nodes proved highly effective in delineating the course of the TD during thoracoscopic esophagectomy, reducing the risk of postoperative chylothorax (13). Likewise, this approach has been successfully employed to detect the leakage point in patients with idiopathic recurrent chylothorax (14). In the present experience, ICG was employed to identify the lymphatic leakage point following mediastinal lymphadenectomy. The application of this technique has already been described in a similar context: in accordance with our findings, Yang et al. reported a 100% success rate in a series of four patients who developed a high-output chylothorax after lung surgery with lymphadenectomy (7). However, contrarily to the abovementioned experiences, we employed a last-generation device that allowed an overlay of fluorescent light images and a well-lighted operative field (**Figures 3 and 4**). In our opinion, this further improved the recognition of the leakage point and enabled a precise ligation of the lymphatic structures under a direct fluorescence guide.

In regard to the ICG technique, with the aim of rapidly outlining the TD, our institutional approach is to instil the dye directly into groin lymph nodes. Nonetheless, other authors reported that a subcutaneous ICG may yield similar results in terms of TD delineation (7), although to guarantee an acceptable TD opacification, some clinicians administer the injection 12–18 h before the scheduled surgery (15). However, the kinetics of ICG absorption into the lymphatic system is beyond the scope of this report, but this will be the object of future research.

In our limited experience in this study, we did not observe any clinical event that might be interpreted as an adverse reaction to ICG. In this sense, our experience is consistent with that of other series wherein ICG was administered intravenously (7, 13). After endovenous administration in a pediatric population at a dose of 0.1–0.2 mg/kg, adverse reactions seem to be limited to mild allergic skin reactions (16), while its use in the adult population at a concentration of 0.25 mg/kg does not seem to be associated with ICG-specific adverse events (17). However, it should be noted that the safety profile of this dye is far from being established due to the low level of information in this regard.

From our point of view, fluorescence-guided identification of the TD represents an optimal choice in this setting for several reasons, some of which are given below:

• intralymphatic injection of ICG is burdened with a low profile of adverse events;
• it allows a rapid layout of the anatomy of the TD and its tributaries, and it clearly points out the leakage point even in the presence of traumatized tissues;
• it gives an immediate feedback on the effectiveness of ligation, reducing the risk of unnecessary further surgical manipulations and promoting an earlier restart of oral feeding, with consequent advantages in terms of admission times and overall costs.

## Limitations

Given the low number of patients and the kind of reporting required by this study, our results should be interpreted with caution and possibly confirmed by larger investigations. The reported success in identifying the TD with fluorescence in our experience does not imply that the procedure may be effective in all cases. However, our intention was to describe a supplementary tool to increase the chances of success when operating patients with postoperative chylothorax. In addition, even though ICG seems to have a quite favorable safety profile, very limited information is available pertaining to the incidence of adverse events post intranodal/subcutaneous injection. In our opinion, this aspect should be discussed with the patient when offering this treatment.

## Conclusion

In our limited experience in this study, we demonstrate the use of ICG as a safe and effective adjuvant for the surgical treatment of postoperative chylothorax. However, studies involving a larger population data set are needed to check the veracity of our findings. Further investigations should assess the role of this procedure in the chylothoraces of other etiologies and whether it can act as a tool for the prevention of chylothorax in high-risk patients when lung resections with extended lymphadenectomy are planned.

## Data Availability Statement

No dataset was generated for the present study. However, data supporting our findings are available from the corresponding author upon reasonable request.

## Ethics Statement

Ethical review and approval was not required for the study on human participants in accordance with the local legislation and institutional requirements. The patients/participants provided their written informed consent to participate in this study.
